# Carbohydrate Counting App Using Image Recognition for Youth With Type 1 Diabetes: Pilot Randomized Control Trial

**DOI:** 10.2196/22074

**Published:** 2020-10-28

**Authors:** Jeffrey E Alfonsi, Elizabeth E Y Choi, Taha Arshad, Stacie-Ann S Sammott, Vanita Pais, Cynthia Nguyen, Bryan R Maguire, Jennifer N Stinson, Mark R Palmert

**Affiliations:** 1 Department of Medicine Schulich School of Medicine & Dentistry Western University London, ON Canada; 2 Inner Analytics Inc Toronto, ON Canada; 3 Division of Endocrinology Hospital for Sick Children Toronto, ON Canada; 4 Peter Gilgan Centre for Research and Learning The Hospital for Sick Children Toronto, ON Canada; 5 Biostatistics Design and Analysis Unit SickKids Research Institute Toronto, ON Canada; 6 Lawrence S Bloomberg Faculty of Nursing University of Toronto Toronto, ON Canada; 7 Departments of Pediatrics and Physiology University of Toronto Toronto, ON Canada

**Keywords:** carbohydrate counting, type 1 diabetes, image recognition, youth, digital health applications (apps), mHealth

## Abstract

**Background:**

Carbohydrate counting is an important component of diabetes management, but it is challenging, often performed inaccurately, and can be a barrier to optimal diabetes management. iSpy is a novel mobile app that leverages machine learning to allow food identification through images and that was designed to assist youth with type 1 diabetes in counting carbohydrates.

**Objective:**

Our objective was to test the app's usability and potential impact on carbohydrate counting accuracy.

**Methods:**

Iterative usability testing (3 cycles) was conducted involving a total of 16 individuals aged 8.5-17.0 years with type 1 diabetes. Participants were provided a mobile device and asked to complete tasks using iSpy app features while thinking aloud. Errors were noted, acceptability was assessed, and refinement and retesting were performed across cycles. Subsequently, iSpy was evaluated in a pilot randomized controlled trial with 22 iSpy users and 22 usual care controls aged 10-17 years. Primary outcome was change in carbohydrate counting ability over 3 months. Secondary outcomes included levels of engagement and acceptability. Change in HbA_1c_ level was also assessed.

**Results:**

Use of iSpy was associated with improved carbohydrate counting accuracy (total grams per meal, *P*=.008), reduced frequency of individual counting errors greater than 10 g (*P*=.047), and lower HbA_1c_ levels (*P*=.03). Qualitative interviews and acceptability scale scores were positive. No major technical challenges were identified. Moreover, 43% (9/21) of iSpy participants were still engaged, with usage at least once every 2 weeks, at the end of the study.

**Conclusions:**

Our results provide evidence of efficacy and high acceptability of a novel carbohydrate counting app, supporting the advancement of digital health apps for diabetes care among youth with type 1 diabetes. Further testing is needed, but iSpy may be a useful adjunct to traditional diabetes management.

**Trial Registration:**

ClinicalTrials.gov NCT04354142; https://clinicaltrials.gov/ct2/show/NCT04354142

## Introduction

Type 1 diabetes is among the most common chronic diseases of childhood, and its incidence is rising [1]. The management of diabetes in youth is complex and impacted by numerous factors including numeracy skills, education, socioeconomic status, family dynamics, engagement with treatment regimens, and use of technologies such as pumps and continuous sensors. Among these factors, insulin administration remains the cornerstone of type 1 diabetes management, but its optimal dosing is often complicated by the need to count carbohydrates [2-4]. Carbohydrate counting allows individuals with type 1 diabetes to match their insulin doses to planned food consumption, and accurate carbohydrate counting can improve blood glucose control (measured by hemoglobin A_1c_; HbA_1c_) [2]. For example, in one study [5] focused on parents of children with type 1 diabetes, more accurate parental carbohydrate counting was associated with 0.8% lower HbA_1c_ values in their children. Among adults with type 1 diabetes, a meta-analysis [6] of 5 studies showed that HbA_1c_ levels improved by an average of 0.6% with improved carbohydrate counting.

Despite its importance, up to two-thirds of individuals with diabetes report having trouble with carbohydrate counting [7]. It has been reported that only a quarter of youth can routinely count carbohydrates within 10 g of the true net carbohydrate value, even for commonly eaten foods [8] and that carbohydrate counting is a barrier to diabetes management [9]. Carbohydrate counting often requires multiple training sessions with experienced dietitians or educators and ongoing efforts by patients and families to maintain competency. Estimating carbohydrate intake can be difficult when the portions of food being consumed are not the same as those listed in an exchange system or on the food label, requiring youth to adjust the carbohydrate count to the appropriate portion size. Accuracy of carbohydrate counting can be further limited by low nutritional literacy and poor numeracy skills [10].

Technologies such as mobile health apps that address these barriers have the potential to ease burden and improve blood glucose control. Unfortunately, most diabetes-related mobile health apps have not undergone formal evaluation and lack evidence of clinical effectiveness, making it difficult for prospective users to assess the value of a particular app to their self-management [11], a situation that is also true within the domain of carbohydrate counting apps.

To help address these gaps, a mobile app was designed and developed to assist youth in counting carbohydrates. The app (iSpy) uses image recognition and artificial intelligence to identify foods and report their carbohydrate content. Here we addressed the question of how iSpy would perform during usability and pilot testing. We hypothesized that it would be well-accepted and that its use would be associated with improved carbohydrate counting accuracy.

## Methods

### Description of iSpy

The iSpy app (see Multimedia Appendix 1 for screenshots) was developed and evaluated in sequential phases [12,13]. The image recognition algorithm that identifies foods from images uses a convolutional neural network. The interface for iSpy was initially co-designed with intended users including certified diabetes educators, registered dietitians, and individuals (aged 12-75 years) living with type 1 diabetes. Once developed, cycles of refinement were conducted after assessing how users functionally navigated the app. The app was then tested for accuracy on a sample of 200 commonly consumed food items (169 items from the Youth Adolescent Food Frequency Questionnaire [14] and 31 commonly consumed complex items containing 2 or more components) selected by a registered dietitian and diabetes educator. An accuracy test required iSpy to report a carbohydrate content that was within 10 g of the food item’s true net (total minus fiber) carbohydrate value [15]. Revisions were made until iSpy was able to achieve this degree of accuracy for ≥90% (180/200) of the items. Current overall accuracy is 94.5% (189/200). The app was then moved into clinical testing, described herein.

### Setting

The usability testing and pilot randomized controlled trial were approved by the research ethics board and conducted within the diabetes program at The Hospital for Sick Children. Informed consent was obtained from all participants, and the pilot randomized controlled trial was registered with clinicaltrials.gov (NCT04354142).

### Usability Testing Procedures

Inclusion criteria were (1) age 8.0-18.0 years, (2) a diagnosis of type 1 diabetes per Diabetes Canada guidelines [16], (3) use of carbohydrate counting as part of treatment regimen, and (4) fluency in English (iSpy is only available in English). The sole exclusion criterion was cognitive impairments.

Iterative cycles of testing and app refinement (3 cycles) were utilized. Testing consisted of 4 scenario-based tasks that were developed using standardized guidelines [17], a semistructured interview, and app acceptability measured by the 5-point Acceptability E-Scale [13]. The focus for the task was on user performance (ie, ease of use, navigation among screens, functions, errors, and efficiency); the semistructured interview and Acceptability E-Scale were focused on overall satisfaction with the app. Participants were purposively selected to achieve a range of age, gender, and duration of type 1 diabetes. The participants were asked to think aloud during use of the app and dialog was audiorecorded.

Participants were provided with an Android or iOS mobile device, depending on the participant’s preference. Scenario-based tasks included use of app features such as photo taking, portion sizing, and food identification. Errors, efficiency (time taken to complete a task), acceptability (ease of use), and suggestions for improvements were logged, and tasks were classified into 1 of 3 categories (successfully completed, completed with minor issues, incomplete due to usability issues). Following each cycle, refinements were made to the user interface based on problems and recommendations, with the revised interface being evaluated in the subsequent cycle [13]. iSpy was moved to pilot testing (pilot randomized controlled trial) when no further issues were identified in the third cycle.

### Pilot Randomized Controlled Trial Procedures

Inclusion criteria were (1) age 10 years-17.0 years (adjusted after usability testing because those under 10 years of age had difficulty navigating the app), (2) ≥6 months since diagnosis with type 1 diabetes, (3) completion of initial carbohydrate counting classes, (4) incorporation of carbohydrate counting into treatment regimen, and (5) access to a smartphone and data plan. Exclusion criteria were (1) cognitive impairment, (2) comorbid physical or psychiatric conditions that might impact ability to use iSpy, (3) diagnosis of a condition that affects dietary exposure, and (4) participation in usability testing.

A convenience sample was enrolled (n=46) and randomly assigned to either usual care (control) or usual care and iSpy (intervention) group using a 2-group randomized block design in blocks of 4 and 6, where the block sizes were not known to the investigator. The randomization schedule was created using SAS (version 9.4; SAS Institute). Data from previous work in our clinic [18] was used to estimate the sample size, indicating that 20 participants per group would be sufficient to detect a mean accuracy difference of 7.1 g in carbohydrate counting (which fit with our aim of assessing accuracy within 10 g), assuming 80% power (β=.2), α=.05, and using a 2-sided paired *t* test; therefore, 23 participants were recruited per group to allow for potential dropout over the 3-month trial.

Duration of diabetes (time since diagnosis) and HbA_1c_ levels were obtained from chart review. Accuracy and efficiency (time taken) of carbohydrate counting were based on a performance task. Participants counted carbohydrates for 10 foods (consisting of 2 foods from each of the 4 main food groups—vegetables and fruit, grain products, milk and alternatives, and meat and alternatives—and in addition, desserts). In each of the 5 food groups, a simple food item (eg, a single item such as an apple) as well as a complex food item (eg, an item containing 2 or more components but with the base food from the selected food group, such as pasta with tomato sauce) were included. Two sets of foods (Diet A and Diet B) of similar difficulty were utilized with half of the participants in each group counting foods from Diet A at baseline and foods from Diet B at 3 months, and vice versa for the other half. This methodology allowed us to control for confounding from participants educating themselves on test items or from any unanticipated differences between test diets. The net carbohydrate value for each food item was determined by either the nutrition label for packaged foods, the United States Department of Agriculture’s National Nutrient Database for Standard Reference [19], the Canadian Nutrient File [20], or by our dietitian (VP) who specializes in diabetes care. We chose to utilize the performance task metric to assess carbohydrate counting instead of using tools such as the PedCarbQuiz [21] so that we could assess the effect of iSpy on counting the carbohydrate content of foods as opposed to its effect on domains such as nutrition label reading or insulin dosing, which are part of the PedCarbQuiz.

Additional measures were also collected. At baseline, comfort with technology was assessed. Quality of life, measured by a subset of questions from quality of life for youth [22,23]; self-care, measured by a subset of questions from the Self Care Inventory [24-26]; and patient or parent responsibility, measured by a subset of questions from Diabetes Family Responsibility Questionnaire were also assessed at baseline and 3 months postintervention. We also assessed factors related to usability of the app including fidelity (tracking of technical difficulties, errors within the app); levels of engagement; and acceptability using a 7-item Acceptability E-Scale (5-point scale) [27]. Qualitative feedback was obtained via postintervention, semistructured interviews among all iSpy users.

At the start of the study, participants in the intervention group downloaded the app on their phone, and a demonstration of iSpy and its functionality were provided. iSpy participants were instructed to use the app at their discretion and when they thought its use would be beneficial. We recognized, for example, that participants may know the carbohydrate counts of the food items that they regularly consume. Thus, they may only want to use iSpy occasionally to assess the counts of only some of these food items whereas they may want to use the app more frequently for food items that they do not regularly consume. Given these instructions instead of a recommended number of uses per day, engagement levels were assessed based on frequency of using the app to log foods per week categorized as high (logging ≥2 meals per week), medium (logging ≥1 meal every 2 weeks but <2 times per week), or low (logging <1 meal every 2 weeks). This structure is similar to that used by others to assess app use [28]. In other instructions, iSpy participants were asked to contact the team should they encounter technical difficulties, and they received a phone call 6 weeks postbaseline for general troubleshooting. As this was a pilot study, we strove to encourage the use of iSpy by sending a maximum of 3 automated alerts to participants not accessing iSpy at least once every 2 weeks.

Statistical analysis for the pilot randomized controlled trial was conducted using R (version 3.6.0) statistical software. Descriptive statistics of participant characteristics for the intervention and control groups are presented as means and standard deviations for continuous variables, and counts and proportions for categorical variables. Differences in these characteristics between the intervention and control groups were tested using 2-sided independent *t* tests for continuous variables, and chi-square tests for categorical variables.

Differences between the intervention and control group on the primary outcome variables (accuracy, time taken for counting, and the percentage of food items for which participants estimated the carbohydrate content within 10 g of the true net carbohydrate value), secondary outcomes (quality of life for youth, self-care, and patient or parent responsibility), and HbA_1c_ level at baseline were examined using 2-sided independent *t* tests. Differences in these variables between the intervention and control group at the follow-up visit were assessed using multiple linear regression models, which included the baseline as a covariate. *P* values <.05 were considered to be statistically significant.

## Results

### Usability Testing

**Figure 1:**
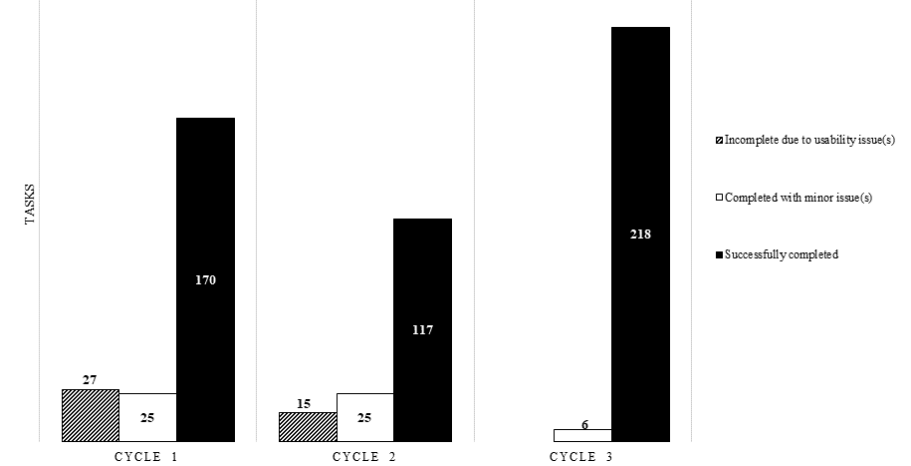
Usability testing errors per cycle representing tasks completed during each cycle of usability testing. The total number of tasks varied per cycle (cycle 1: 222; cycle 2: 157; cycle 3: 224).

Youth (total: n=16—cycle 1: n=6; cycle 2: n=4, cycle 3: n=6) ranging in age from 8.5 to 17.0 years (mean 13.5, SD 2.6 years) participated in iSpy’s iterative usability testing. Scenarios consisting of multiple tasks were used; based on how the participant responded to iSpy or how image recognition classified the food within each scenario, follow-up tasks were required, with the total number of tasks across 4 scenarios varying between 35 and 41 per participant. Errors within each cycle were tracked (Figure 1). In cycle 1, a total of 27 errors preventing successful completion of tasks occurred (mean 4.5 SD 4.4 per participant), representing 12.2% (27/222) of the total tasks. In response, modifications were made such as simplifying the user interface and changing wording so that the app flow was more intuitive. In cycle 2, errors were made on 9.6% of the tasks (15/157). Additional changes were made to the app including simplifying input requirements, making only one action possible at a time, improving graphics, and clarifying instructions. In cycle 3, no errors (0/224, 0%) preventing task completion occurred, and only 2.7% of tasks (6/224) had minor incidents. Acceptability E-Scale scores were positive (mean 4.6, SD 0.7) on domains that included helpfulness in carbohydrate counting and food identification, ease of use, time taken, and overall satisfaction across all 3 cycles of testing. Postcycle 3, minor modifications such as aesthetic changes to the user interface were made prior to pilot testing (pilot randomized controlled trial).

### Pilot Randomized Controlled Trial

**Figure 2:**
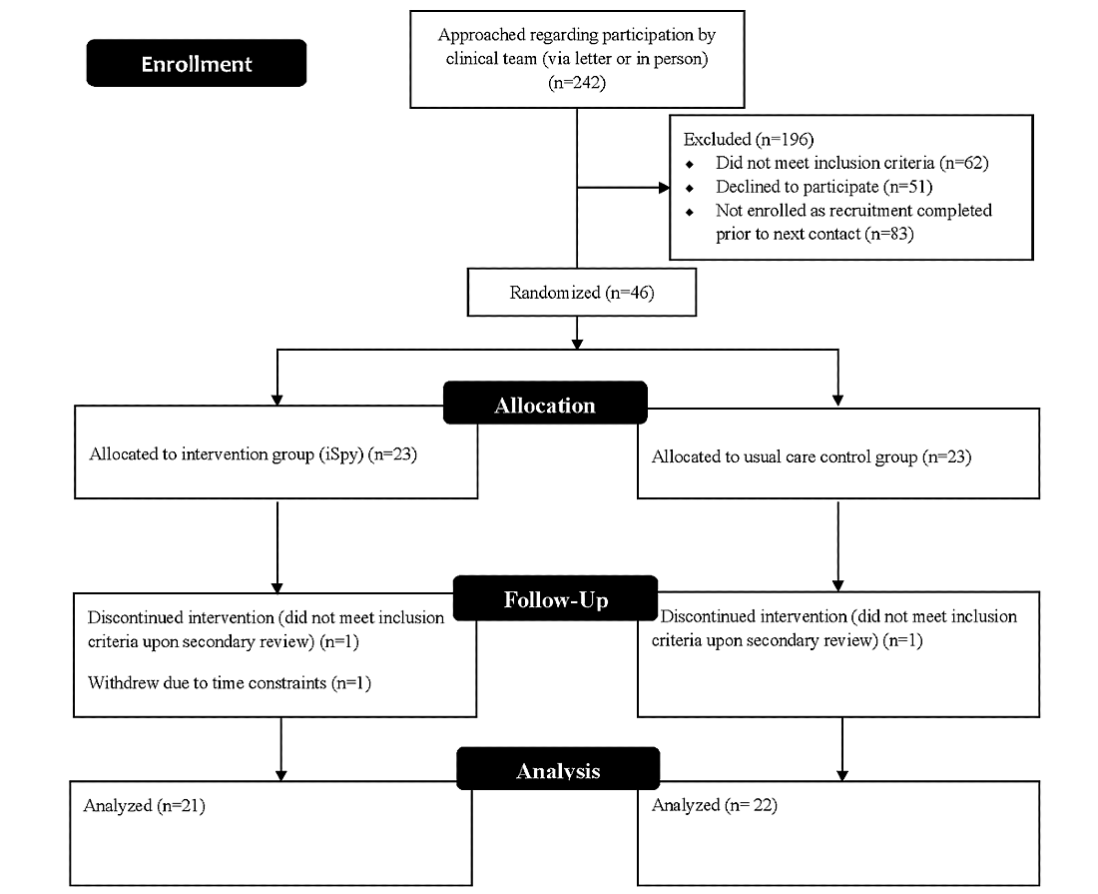
Participant flowchart.

Of the 46 participants who were enrolled and randomly allocated into the 2 arms of the pilot study, 43 participants completed the study (Figure 2). All participants reported being comfortable using computers and smart devices, and there were no significant differences between the 2 groups for any of the baseline characteristics (gender: *P*=.22; age: *P*=.99; duration since diagnosis: *P*=.79; regulation method: *P*=.62; confidence in counting: *P*=.39; Table 1).

At baseline, there was also no difference in carbohydrate counting accuracy or time taken to complete the task between the 2 groups. At the 3-month follow-up visit, the iSpy group displayed a statistically significant increase in carbohydrate counting accuracy (*P=*.008), and a statistically significant decrease in counting errors (*P=*.047) compared to that of the control group (Table 2). None of the secondary outcome variables such as quality of life measures (*P=*.64), self-care measures (*P=*.17), or patient/parent responsibility (*P=*.69), differed between the groups at baseline and 3-month postintervention period. Although not a main outcome variable for this pilot study, HbA_1c_ values were assessed at baseline and at the 3-month follow-up visit, with the iSpy group displaying statistically significant lower HbA_1c_ values (*P=*.03) compared to those of the control group.

**Table 1:** Participant characteristics (at baseline).

Characteristics		iSpy intervention (n=22)		Usual care (n=22)		*P* value	
**Gender, n (%)**						.22	
	Male		11 (50)		16 (73)		
	Female		11 (50)		6 (27)		
Age (in years), mean (SD)		13.98 (1.57)		13.98 (1.76)		.99	
Duration since diagnosis (in years), mean (SD)		6.08 (4.14)		6.44 (4.45)		.79	
**Method of insulin regulation, n (%)**						.62	
	Pump		11 (50)		10 (46)		
	Other		11 (50)		12 (54)		
Rated confidence in carbohydrate counting skills (out of 10), mean (SD)		6.70 (1.59)		7.24 (2.30)		.39	

**Table 2:** Carbohydrate counting and glycemic control outcomes (at baseline and follow-up).

Outcomes	Baseline			Follow-up			Coefficient
	iSpy (n=22)	Usual care (n=22)	*P* value	iSpy (n=21)	Usual care (n=22)	*P* value	
Absolute error (% of total grams), mean (SD)	31.97 (11.36)	32.03 (10.01)	.99	27.45 (10.90)	38.00 (14.74)	.008	–10.479
Errors >10 g (%), mean (SD)	25.00 (14.06)	27.73 (15.10)	.54	21.43 (16.82)	32.27 (16.31)	.047	–9.851
Total time (seconds), mean (SD)	79.95 (23.88)	80.73 (27.82)	.92	74.86 (31.78)	78.23 (44.97)	.79	–2.741
HbA_1c_^a^ (%), mean (SD)	8.41 (1.84)	8.35 (1.32)	.91	8.06 (1.43)	8.80 (1.60)	.03	–0.603

^a^HbA_1c_: hemoglobin A_1c_.

**Figure 3:**
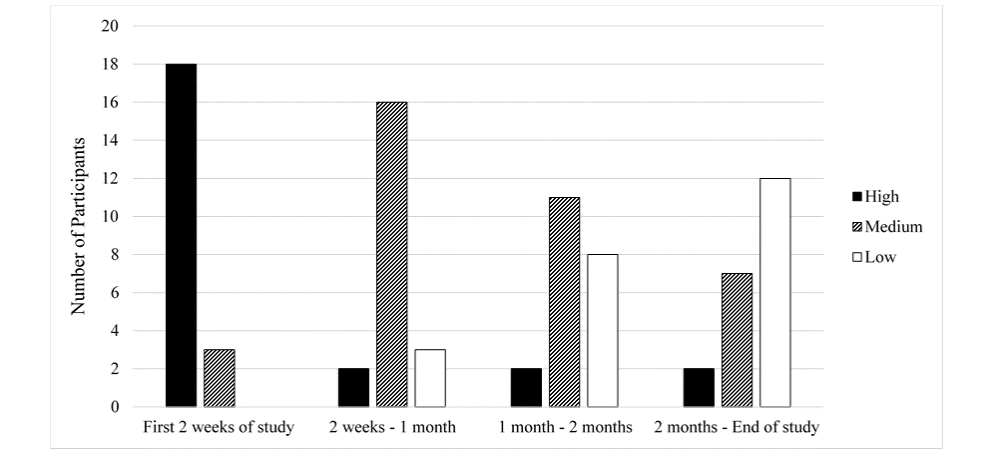
Engagement levels with the number of participants in each category displayed for each time frame.

No major technical challenges were identified. App engagement was assessed over 4 time periods (first 2 weeks of study, 2 weeks to 1 month, first to second month, and second month to end of study), with 43% (9/21) of participants indicating medium or high use at the end of study (Figure 3). Over the course of the study, a mean of 1.9 (SD 0.94) reminder emails were sent to iSpy users.

Acceptability E-Scale results were positive (Multimedia Appendix 2). Of the 7 questions asked, iSpy respondents ranked iSpy positively in 6 out of 7 categories. The highest rankings were related to ease of understanding and ease of use. The weakest responses were related to how helpful iSpy was in food identification.

Semistructured interviews were conducted among all iSpy participants, and results mirrored those of the questionnaires. Most participants (18/21, 86%) found the iSpy app fairly to very easy to use. Participants preferred using the photos followed by text features to identify foods with few respondents utilizing the voice function. Participants also valued speed of image recognition results and delays due to misidentification of foods were viewed negatively. Participants provided suggestions for improvement, such as including additional options for portion sizing, refining the identification features so that foods within a complex meal do not need to be added one-by-one, expanding the database of known foods, and including optional reminder notifications about logging foods.

## Discussion

### Principal Results

With few exceptions [28,29], apps used to facilitate diabetes care have not undergone formal testing [11,30] making it difficult to assess their utility and limiting the advancement of digital health apps for diabetes care. Here we report iterative usability testing and pilot testing of iSpy showing that use of the app was associated with improved carbohydrate counting accuracy and high acceptability and satisfaction scores. Areas for further refinement were also identified such as increased speed and more focus on image and text recognition features.

Carbohydrate counting is an important component of diabetes care [31-33], and use of iSpy was associated with fewer counting errors of >10 g. Although not measured directly, this degree of improved accuracy would theoretically lead to improved postprandial glucose, and the improvement we observed in HbA_1c_ levels may suggest an effect on overall glycemic control, a finding that warrants further study in future trials. Errors of >10 g are considered clinically important [8,15,34], with one study reporting that children who received prandial insulin boluses based on carbohydrate estimates within 10 g had minimal changes in blood glucose postprandially [34]. On the other hand, when insulin boluses were based on carbohydrate estimates that were off by 20 g, more instances of hypo- and hyperglycemia occurred 2-3 hours after the meal [15].

### Comparison With Prior Work

iSpy is not the only app that has been developed to assist patients or caregivers with carbohydrate counting; however, the majority of the other apps, such as MyFitnessPal or Samsung Health, are general nutrition tracking apps. Data are limited, but available studies report conflicting information regarding the difference between output nutrient data from these apps compared to reference data [35,36]. Furthermore, these apps have limited input modalities (generally limited to manual text searching) [37]. Other diabetes-related apps address multiple aspects of diabetes self-management, including tracking of glucose data, physical activity, diet, and insulin doses; such apps may also include assistance with carbohydrate counting. When tested, many of these apps have not demonstrated significant improvements in their primary outcomes, which have mainly been centered on glycemic control [28,38]. It is also worth noting that while these apps are all-encompassing for diabetes self-management, some recommend educational features such as carbohydrate counting to improve their usage [39]. However, apps that have been developed specifically to assist with carbohydrate counting are limited, and few of these have been formally evaluated, with studies having only been conducted over a duration of a few days or weeks and lacking successful comparisons with controls [40-44].

Thus, our phased development of iSpy, along with usability testing and the 3-month pilot study are relatively unique, as are the promising results. It is possible that use of iSpy was associated with more accurate carbohydrate counting because iSpy reinforced a structured approach to carbohydrate counting: identifying each food item, determining the portion size being consumed, and asking about any “hidden” carbohydrates, such as barbeque sauce under a bun. Whether this step-by-step approach to carbohydrate counting with real-time feedback underlies the observed improvement can be tested in subsequent studies.

Participant engagement is an additional measure of an app’s usability. Although use was declining, at the end of the 3-month trial 9/21 (43%) participants were still medium to high users. It is difficult to know how to interpret this degree of usage. One could view this percentage of individuals with use at a minimum of ≥1 meal every 2 weeks as evidence of engagement that is diminishing too rapidly. However, it is our experience that app usage often wanes over time, even when users rate the app quite favorably [28]. It is this expected degree of dropoff that led us to define ongoing usage of at least ≥1 meal every 2 weeks as medium engagement. Moreover, despite the dropoff, iSpy use was associated with improved carbohydrate counting at 3 months, suggesting that use of an educational app may have long lasting impact even after the period of high use has ended. Nevertheless, it will be important to consider options to further improve engagement such as push alert notifications, reminders, and ensuring ease of data entry.

Although randomized controlled trials are considered the gold standard for evaluating efficacy, there is concern that such trials may not be optimal for the assessment of apps. Thus, when considering future testing of iSpy and other apps, one must acknowledge the long timeline for recruitment and conduct of such trials within a rapidly and continually evolving technology-based environment [28,45]. User testing and product revision often occurs on shorter timelines, necessitating consideration of adaptive clinical trials that allow for continual modifications while data are being collected [46]. Furthermore, evaluation of potential barriers to incorporating app use into ongoing clinical care should also be assessed as a component of such trials. Assessing and addressing topics such as workflow integration and patient (or family)–provider communication will be needed to continue to support effective advancement of digital health [28,45].

### Limitations

While our results are encouraging, we acknowledge that our studies may have had some limitations. The studies were conducted at a single tertiary pediatric center, and the results may not be generalizable. A larger trial and wider clinical implementation study is an important next step to verify our findings. In addition, although based on databases of commonly consumed foods [14], the number of foods recognized by iSpy is not all-encompassing. The database was not identified as a limiting factor by our participants, but we will continue to expand iSpy’s ability to recognize foods eaten around the world among different cultures. Though no differences were found between the intervention and control groups for baseline technology familiarity and use, we did not acquire detailed information about other factors that can influence care such as education level, socioeconomic status data, family dynamics, or details of treatment regimen, all of which could have accounted for some differences. Finally, we did not provide text reminders to the control subjects in this pilot study. Although these reminders were brief texts that occurred at most 3 times over the trial, it is feasible that they could have motivated change and thus represent a confounding variable affecting our results.

### Conclusion

Carbohydrate counting remains a challenge for youth with type 1 diabetes and their families, and errors in counting can have clinical impact. We have developed and conducted rigorous pilot testing of an app designed to assist youth with carbohydrate counting. The data suggest that use of iSpy is associated with improved carbohydrate counting and that usability and acceptability of the app is quite positive. Further testing is now warranted to verify these pilot data and determine if the app can indeed improve blood glucose control and help decrease the burden of living with type 1 diabetes.

## Appendix

Multimedia Appendix 1 Screenshots of the primary features of the iSpy app.

Multimedia Appendix 2 Acceptability e-scale questionnaire results.

## Abbreviations

HbA_1c_: hemoglobin A_1c_

## References

[1] Chapter 5: Diabetes in Canada: facts and figures from a public health perspective – youth and children. Government of Canada Public Health Agency of Canada.

[2] Cheng AYY, Canadian Diabetes Association Clinical Practice Guidelines Expert Committee (2013). Canadian Diabetes Association 2013 clinical practice guidelines for the prevention and management of diabetes in Canada. Introduction. Can J Diabetes.

[3] Gökşen Damla, Atik Altınok Y, Ozen S, Demir G, Darcan S (2014). Effects of carbohydrate counting method on metabolic control in children with type 1 diabetes mellitus. J Clin Res Pediatr Endocrinol.

[4] Brazeau AS, Mircescu H, Desjardins K, Leroux C, Strychar I, Ekoé J M, Rabasa-Lhoret R (2013). Carbohydrate counting accuracy and blood glucose variability in adults with type 1 diabetes. Diabetes Res Clin Pract.

[5] Mehta SN, Quinn N, Volkening LK, Laffel LMB (2009). Impact of carbohydrate counting on glycemic control in children with type 1 diabetes. Diabetes Care.

[6] Bell KJ, Barclay AW, Petocz P, Colagiuri S, Brand-Miller JC (2014). Efficacy of carbohydrate counting in type 1 diabetes: a systematic review and meta-analysis. Lancet Diabetes Endocrinol.

[7] Ahola AJ, Mäkimattila Sari, Saraheimo M, Mikkilä Vera, Forsblom C, Freese R, Groop P, FinnDIANE Study Group (2010). Many patients with Type 1 diabetes estimate their prandial insulin need inappropriately. J Diabetes.

[8] Bishop FK, Maahs DM, Spiegel G, Owen D, Klingensmith GJ, Bortsov A, Thomas J, Mayer-Davis EJ (2009). The Carbohydrate Counting in Adolescents With Type 1 Diabetes (CCAT) Study. Diabetes Spectrum.

[9] Lancaster BM, Pfeffer B, McElligott M, Ferguson AT, Miller M, Wallace D, Lane JT (2010). Assessing treatment barriers in young adults with type 1 diabetes. Diabetes Res Clin Pract.

[10] White RO, Wolff K, Cavanaugh KL, Rothman R (2010). Addressing health literacy and numeracy to improve diabetes education and care. Diabetes Spectr.

[11] Eng DS, Lee JM (2013). The promise and peril of mobile health applications for diabetes and endocrinology. Pediatr Diabetes.

[12] Danaher BG, Seeley JR (2009). Methodological issues in research on web-based behavioral interventions. Ann Behav Med.

[13] Campbell M, Fitzpatrick R, Haines A, Kinmonth AL, Sandercock P, Spiegelhalter D, Tyrer P (2000). Framework for design and evaluation of complex interventions to improve health. BMJ.

[14] Rockett HR, Breitenbach M, Frazier AL, Witschi J, Wolf AM, Field AE, Colditz GA (1997). Validation of a youth/adolescent food frequency questionnaire. Prev Med.

[15] Smart CE, King BR, McElduff P, Collins CE (2012). In children using intensive insulin therapy, a 20-g variation in carbohydrate amount significantly impacts on postprandial glycaemia. Diabet Med.

[16] Sellers Elizabeth, Diabetes Canada Clinical Practice Guidelines Expert Committee (2018). Definition, classification and diagnosis of diabetes, prediabetes and metabolic syndrome. Diabetes Canada 2018 Clinical Practice Guidelines for the Prevention and Management of Diabetes in Canada.

[17] Usability testing. Usability.gov.

[18] Pais V, Patel BP, Ghayoori S, Hamilton JK (2020). Counting carbs to be in charge: a comparison of an internet-based education module with in-class education in adolescents with type 1 diabetes. Clinical Diabetes.

[19] National database for standard reference release 28. The United States Department of Agriculture.

[20] Canadian nutrient file. Government of Canada.

[21] Koontz MB, Cuttler L, Palmert MR, O'Riordan M, Borawski EA, McConnell J, Kern EO (2010). Development and validation of a questionnaire to assess carbohydrate and insulin-dosing knowledge in youth with type 1 diabetes. Diabetes Care.

[22] Ingersoll GM, Marrero DG (1991). A modified quality-of-life measure for youths: psychometric properties. Diabetes Educ.

[23] Skinner TC, Hoey H, McGee HM, Skovlund SE, Hvidøre Study Group on Childhood Diabetes (2006). A short form of the Diabetes Quality of Life for Youth questionnaire: exploratory and confirmatory analysis in a sample of 2,077 young people with type 1 diabetes mellitus. Diabetologia.

[24] La Greca AM, Swales T, Klemp S, Madigan S (1988). Self care behaviors among adolescents with diabetes.

[25] Greco P, La Greca AM, Ireland A, Wick P, Freeman C, Agramonte R (1990). Assessing adherence in IDDM: A comparison of two methods.

[26] Lewin AB, LaGreca AM, Geffken GR, Williams LB, Duke DC, Storch EA, Silverstein JH (2009). Validity and reliability of an adolescent and parent rating scale of type 1 diabetes adherence behaviors: the Self-Care Inventory (SCI). J Pediatr Psychol.

[27] Wu W, Johnson R, Schepp KG, Berry DL (2011). Electronic self-report symptom and quality of life for adolescent patients with cancer: a feasibility study. Cancer Nurs.

[28] Goyal S, Nunn CA, Rotondi M, Couperthwaite AB, Reiser S, Simone A, Katzman DK, Cafazzo JA, Palmert MR (2017). A mobile app for the self-management of type 1 diabetes among adolescents: a randomized controlled trial. JMIR Mhealth Uhealth.

[29] Cafazzo JA, Casselman M, Hamming N, Katzman DK, Palmert MR (2012). Design of an mHealth app for the self-management of adolescent type 1 diabetes: a pilot study. J Med Internet Res.

[30] Veazie S, Winchell K, Gilbert J, Paynter R, Ivlev I, Eden K, Nussbaum K, Weiskopf N, Guise J, Helfand M (2018). Mobile Applications for Self-Management of Diabetes. AHRQ Comparative Effectiveness Technical Briefs.

[31] Tascini G, Berioli MG, Cerquiglini L, Santi E, Mancini G, Rogari F, Toni G, Esposito S (2018). Carbohydrate counting in children and adolescents with type 1 diabetes. Nutrients.

[32] Gokosmanoglu F, Onmez A (2018). Influence of flexible insulin dosing with carbohydrate counting method on metabolic and clinical parameters in type 1 diabetes patients. Open Access Maced J Med Sci.

[33] Yamada E, Okada S, Nakajima Y, Bastie CC, Tagaya Y, Osaki A, Shimoda Y, Shibusawa R, Saito T, Ozawa A, Yamada M (2017). Effect of carbohydrate counting using bolus calculators on glycemic control in type 1 diabetes patients during continuous subcutaneous insulin infusion. J Diabetes Investig.

[34] Smart CE, Ross K, Edge JA, Collins CE, Colyvas K, King BR (2009). Children and adolescents on intensive insulin therapy maintain postprandial glycaemic control without precise carbohydrate counting. Diabet Med.

[35] Fallaize R, Zenun Franco R, Pasang J, Hwang F, Lovegrove JA (2019). Popular nutrition-related mobile apps: an agreement assessment against a UK reference method. JMIR Mhealth Uhealth.

[36] Fadhil A (2019). Comparison of Self-monitoring Feedback Data from Electronic Food and Nutrition Tracking Tools. Cornell University: Computer Science - Computers and Society.

[37] Franco RZ, Fallaize R, Lovegrove JA, Hwang F (2016). Popular nutrition-related mobile apps: a feature assessment. JMIR Mhealth Uhealth.

[38] Skrøvseth Stein Olav, Årsand Eirik, Godtliebsen F, Joakimsen RM (2015). Data-driven personalized feedback to patients with type 1 diabetes: a randomized trial. Diabetes Technol Ther.

[39] Jeffrey B, Bagala M, Creighton A, Leavey T, Nicholls S, Wood C, Longman J, Barker J, Pit S (2019). Mobile phone applications and their use in the self-management of Type 2 Diabetes Mellitus: a qualitative study among app users and non-app users. Diabetol Metab Syndr.

[40] Mookherji S, Mehl G, Kaonga N, Mechael P (2015). Unmet Need: Improving mHealth Evaluation Rigor to Build the Evidence Base. J Health Commun.

[41] Rhyner D, Loher H, Dehais J, Anthimopoulos M, Shevchik S, Botwey RH, Duke D, Stettler C, Diem P, Mougiakakou S (2016). Carbohydrate estimation by a mobile phone-based system versus self-estimations of individuals with type 1 diabetes mellitus: a comparative study. J Med Internet Res.

[42] Domhardt M, Tiefengrabner M, Dinic R, Fötschl U, Oostingh GJ, Stütz T, Stechemesser L, Weitgasser R, Ginzinger SW (2015). Training of carbohydrate estimation for people with diabetes using mobile augmented reality. J Diabetes Sci Technol.

[43] Rollo ME, Ash S, Lyons-Wall P, Russell AW (2015). Evaluation of a mobile phone image-based dietary assessment method in adults with type 2 diabetes. Nutrients.

[44] Rossi MC, Nicolucci A, Lucisano G, Pellegrini F, Di Bartolo Paolo, Miselli V, Anichini R, Vespasiani G, Study Group Did (2013). Impact of the "Diabetes Interactive Diary" telemedicine system on metabolic control, risk of hypoglycemia, and quality of life: a randomized clinical trial in type 1 diabetes. Diabetes Technol Ther.

[45] Pham Q, Wiljer D, Cafazzo JA (2016). Beyond the randomized controlled trial: a review of alternatives in mHealth clinical trial methods. JMIR Mhealth Uhealth.

[46] Thorlund K, Haggstrom J, Park JJ, Mills EJ (2018). Key design considerations for adaptive clinical trials: a primer for clinicians. BMJ.

