# Supplementary figures and images for: Carbohydrate Counting App Using Image Recognition for Youth With Type 1 Diabetes: Pilot Randomized Control Trial
Source: JMIR Mhealth Uhealth. 2020 Oct 28;8(10):e22074. doi: 10.2196/22074 (PMC7657721; doi:10.2196/22074)

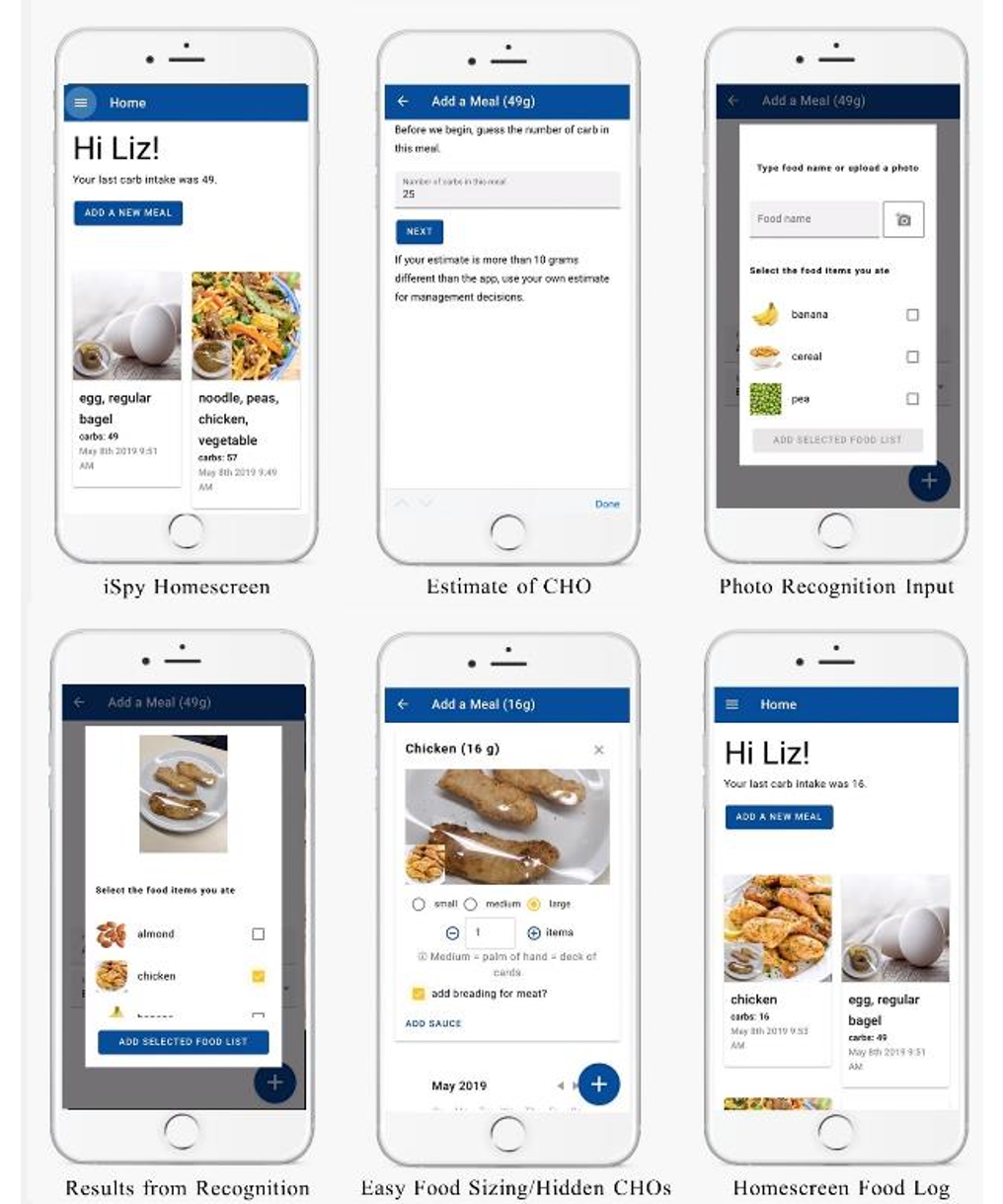

Supplement: Multimedia Appendix 1 [file mhealth_v8i10e22074_app1.png]
