# Supplementary material for: Carbohydrate Counting App Using Image Recognition for Youth With Type 1 Diabetes: Pilot Randomized Control Trial
Source: JMIR Mhealth Uhealth. 2020 Oct 28;8(10):e22074. doi: 10.2196/22074 (PMC7657721; doi:10.2196/22074)
Supplement: Multimedia Appendix 2 [file mhealth_v8i10e22074_app2.docx]

**Acceptability e-scale questionnaire results.**

| Acceptability E-Scale Questionnaire Results | Lower than neutral (% of respondents) | Higher than neutral (% of respondents) |
| --- | --- | --- |
|  |  |  |
| How easy was iSpy for you to use? (1 = Very Difficult, 5 = Very Easy) | 24 | 52 |
| How Understandable Was The Carbohydrate Information Provided By iSpy? (1 = Difficult To Understand, 5 = Easy To Understand) | 19 | 52 |
| How Easy Was It To Understand The Questions iSpy Asked You? (1 = Difficult To Understand, 5 = Easy To Understand) | 10 | 81 |
| How Would You Rate Your Overall Satisfaction With iSpy? (1 = Very Dissatisfied, 5 = Very Satisfied) | 33 | 43 |
| How Helpful Was iSpy In Identifying Your Food? (1 = Very Unhelpful, 5 = Very Helpful) | 48 | 29 |
| How Helpful Was iSpy In Counting Your Carbohydrates? (1 = Very Unhelpful, 5 = Very Helpful) | 29 | 33 |
| Was The Amount Of Time It Took to Complete iSpy Okay? (1 = Very Unacceptable, 5 = Very Acceptable) | 14 | 43 |

Scores below 3 on the 5-point scale are categorized as being “lower than neutral”, and scores above 3 on the 5-point scale are categorized as being “higher than neutral”. Percentages do not sum to 100 because some respondents selected the neutral response.
